# Choroidal metastasis with retinal detachment: A case report

**DOI:** 10.1097/MD.0000000000028009

**Published:** 2021-12-23

**Authors:** Shu-Hua Lin, Yong-Gen Xu, Jing-Hua Zhao, Hong Cui, Hua Jin, Yu-Jie Jia, Jian Zhao, Ying-Jun Li

**Affiliations:** a Department of Ophthalmology, Affiliated Hospital of Yanbian University, Yanji Jilin, China; b Department of Ophthalmology, Peking University Third Hospital, Beijing, China.

**Keywords:** brain metastasis, case report, choroidal metastasis, oesophageal cancer, retinal detachment

## Abstract

**Rationale:**

Breast carcinoma is the most common primary source of choroidal metastasis (CM). In the present case, esophageal cancer was the primary tumour, brain metastasis occurred, and CM occurred later in the left eye with 2 retinal detachments, which is very rare.

**Patient concerns:**

A 62-year-old man complained of a sudden decrease in visual acuity consisting of a small shadow in front of his left eye with a sensation of covered vision after 1 cycle of systemic chemotherapy and radiotherapy for resected esophageal cancer with brain metastasis. Fundus examination revealed exudative retinal detachment without retinal tears. CM with exudative retinal detachment was also considered. The patient refused further treatment. After the second cycle of chemotherapy, there were no significant changes in the retina and visual acuity improved. However, after craniocerebral surgery for brain metastasis, the visual acuity decreased again and showed 3 choroidal masses with macular involvement and retinal detachment but without retinal tears.

**Diagnosis:**

The final diagnosis was CM with retinal detachment.

**Interventions:**

The patient was advised to undergo enucleation of the left eye during the second retinal detachment, but he refused.

**Outcomes:**

Two months after the second retinal detachment, the patient died of systemic metastases.

**Lessons:**

It is important to consider CM when the first retinal detachment and known cancer are diagnosed. At present, it is necessary to develop a standardised treatment plan as well as a multidisciplinary approach to early diagnosis, combined treatment, and timely intervention for such cases.

## 1 Introduction

Oesophageal cancer is the primary malignant tumour of the oesophagus, and squamous cell carcinoma is the most common.^[1]^ The main progress is direct diffusion, lymph node metastasis, and hematogenous metastasis. Approximately 30% of the patients had liver, bone, and lung metastases, and brain metastases ranged from 0.4% to 5.1%.^[2,3]^ Intraocular metastasis is very rare, and due to the lack of lymphatic vessels in the orbital and intraocular tissues, most of the tumour cells enter the eye and spread to the choroid through the blood. Choroidal metastases generally appear as creamy white or pale yellow masses^[4,5]^ and are associated with subretinal fluid, and the prognosis is poor. To the best of our knowledge, this is the first report of esophageal cancer metastasis to the brain and choroid with 2 retinal detachments.

## 2 Case report

**Figure 1:**
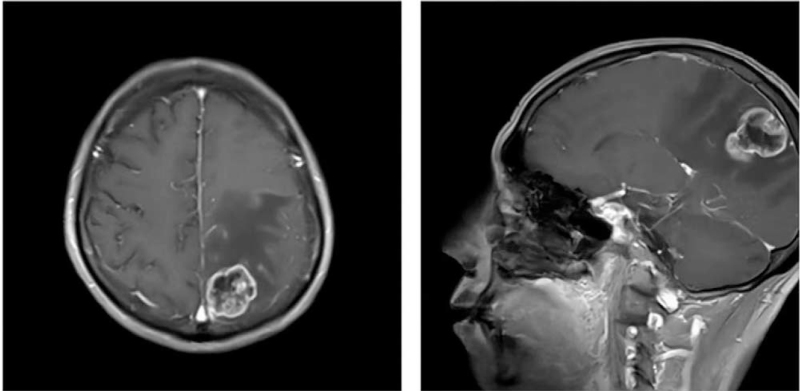
An irregular mass in the left parietal lobe. An uneven T1-weighted low signal and irregular ring enhancement were seen after an enhanced scan of approximately 41 × 26 × 31 mm. The left ventricle was compressed, and the midline structure was shifted to the right.

A 62-year-old man was admitted to our department complaining of a sudden decrease in visual acuity consisting of a small shadow in front of his left eye with the sensation of covered vision after 1 cycle of systemic chemotherapy (cisplatin [CDDP] 75 mg/m^2^d1 + paclitaxel [PTX] 135 mg/m2d1, q 21 d) on November 27, 2019. In his medical history, the patient reported that he had undergone curative resection of oesophageal cancer 2 years ago. The tumour was a squamous carcinoma of T1bN0M0. Brain magnetic resonance imaging (MRI) performed in May 2019 indicated space-occupying lesions in the left parietal lobe (Fig. 1), and whole-brain radiotherapy was performed at a dose of 3 Gy/fraction per day (total dose, 30 Gy).

**Figure 2:**
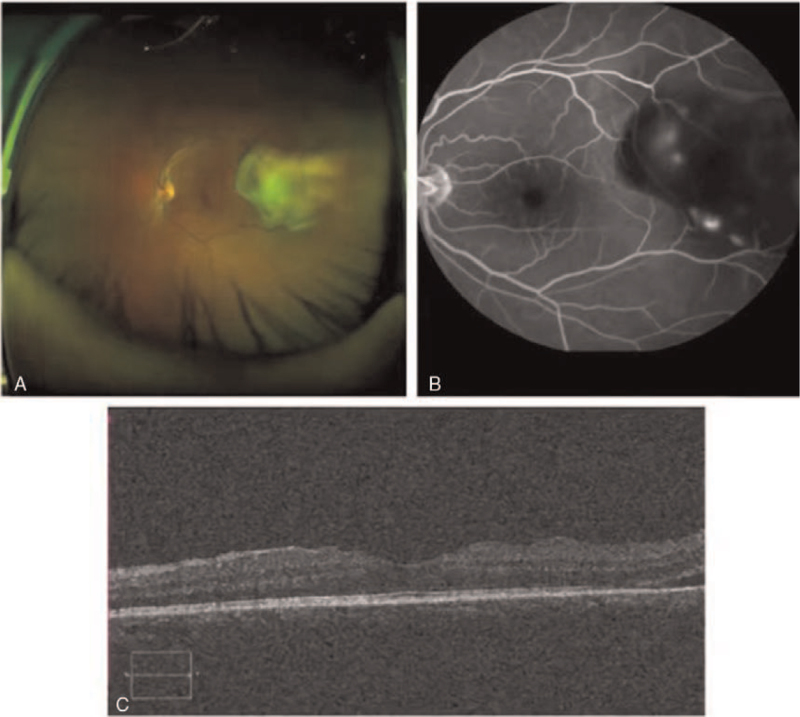
A) Exudative retinal detachment with underlying diffuse, subretinal yellowish-cream-coloured infiltrates. The infiltrates were largely situated on and around the temporal macula, with the periphery being uninvolved. B) Large area of retina capillary without perfusion area and multiple progressive pinpoint foci of hyperfluorescence during the subsequent phase. C) The temporal neuroepithelial layer of macula was slightly detached with effusion.

**Figure 3:**
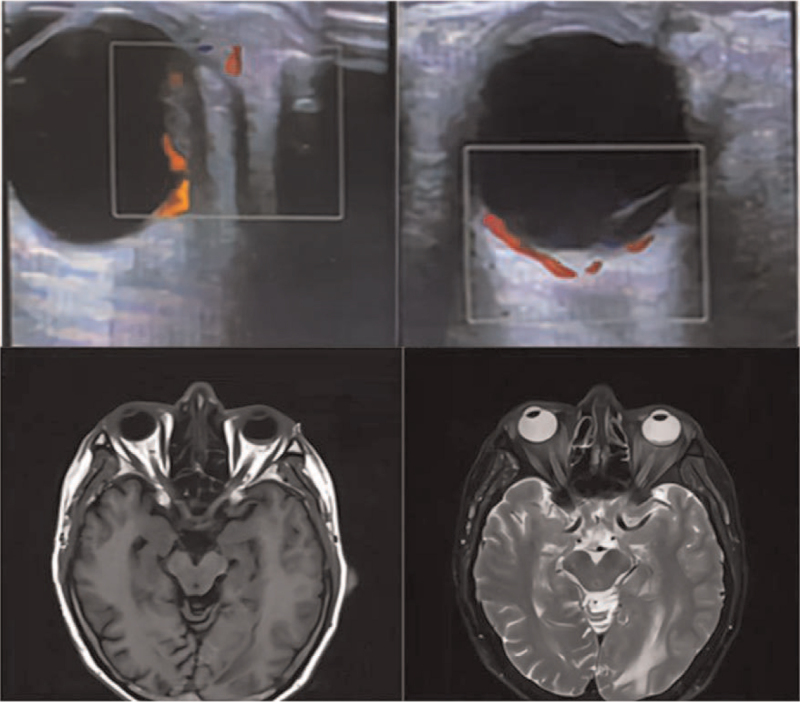
A strong echo of 10 × 4 mm was seen on the temporal side of the vitreous of the left eye and blood flow signals were seen inside. Vitreous membranous echo, no obvious signal inside. MRI: Irregular T1-weighted and T1-weighted low signal shadows were found in the posterior and lateral walls of the left eyeball, protruding into the eyeball.

Distance-corrected visual acuity was 0.1 in the left eye and 1.0 in the right eye, respectively. Anterior segment depth, intraocular pressure, motility, vitreous cavity, and pupillary reflex were normal in both eyes. Ocular fundoscopy was normal in the right eye. In the left eye, fundus examination showed a limited greyish-white eminence of the retina on the temporal side of the macula that was accompanied by exudative retinal detachment, curved retinal vessels on the surface, no clear tears were seen, and there appeared to be a limited choroid eminence under the retina. B-scan ultrasound showed a 10 × 4 mm thin strong echo with tapering edges and retinal detachment. Fundus fluorescein angiography (FFA) showed a large area of retinal capillaries without perfusion area and multiple progressive pinpoint foci of hyperfluorescence during the subsequent phase. Optical coherence tomography revealed that the temporal neuroepithelial layer of the macula was slightly detached from the effusion (Fig. 2). MRI showed irregular T1-weighted and T1-weighted low-signal shadows protruding into the left eyeball from the posterior and lateral walls (Fig. 3).

**Figure 4:**
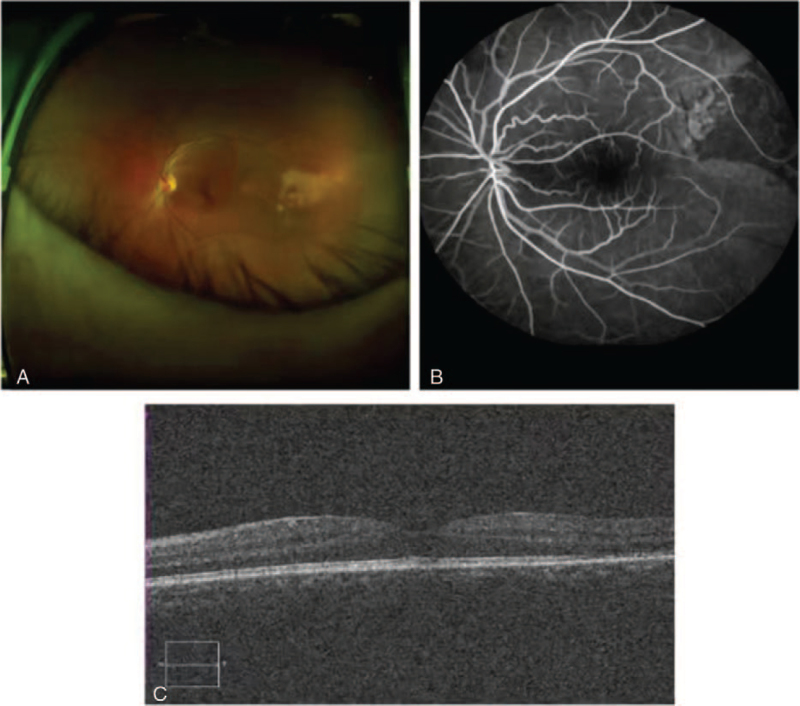
A) After the second cycle of systemic chemotherapy, the subretinal fluid was absorbed, and the retina was restored. B) Loss of retinal pigment epithelium with small vessel leakage. C) The structure of each layer of temporal retinal neuroepithelium of macula is complete and clear.

Choroidal metastasis (CM) with exudative retinal detachment was diagnosed on the basis of characteristic examination and MRI findings, as well as the background of the cancer patient with systemic metastasis. Surgical treatment was not considered, and left eye photodynamic therapy was recommended. The patient refused further treatment and was discharged on the second day. After the second cycle of systemic chemotherapy (CDDP 75 mg/m^2^d1 + PTX 135 mg/m2d1, q 21 d), the patient visited the ophthalmology department for a follow-up visit on December 21, 2019. The patient's visual acuity improved significantly. The distance-corrected visual acuity of the left eye was 0.2, and the intraocular pressure was 15 mm Hg. Fundus examination showed that the subretinal fluid was absorbed, and that the retina was restored. FFA showed loss of the retinal pigment epithelium with small vessel leakage. Optical coherence tomography revealed that the structure of each layer of the temporal retinal neuroepithelium of the macula was complete and clear (Fig. 4). From January 2020 to March 2020, the third cycle of systemic chemotherapy was completed (CDDP 75 mg/m^2^d1 + PTX 135 mg/m2d1, q 21 d) and 2 gamma knife treatments (average marginal tumour doses ranged from 16 Gy to 59 Gy, with an aggregated median tumour dose of 30 Gy [25–36]) were performed for the brain metastasis, and no obvious changes were found in the retina.

**Figure 5:**
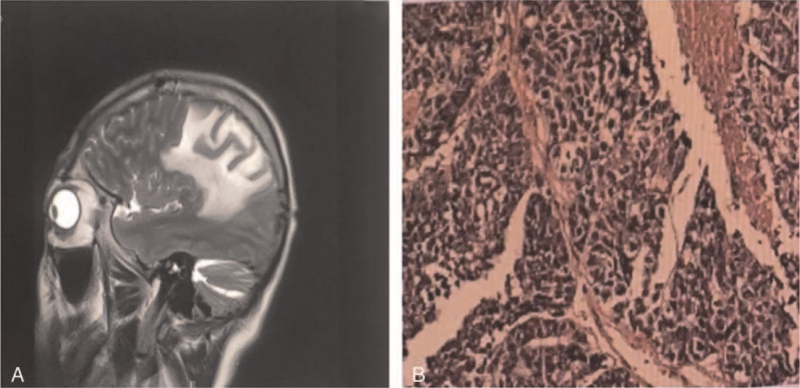
A) Irregular mixed signal clumps (approximately 4.3 × 2.0 cm) can be seen on the left parietal lobe. T1-weighted, T2-FLAIR and DW1 show high and low mixed signal shadows. The left ventricle was compressed, and the midline structure was shifted to the right. B) The space-occupying lesions were metastatic poorly differentiated squamous cell carcinoma. Immuno Histo Chemistry (IHC) 67(+70%), CKpan (+), CD56(−), Syn(−), CgA(−), TTF-1(−), CK5/6(+), LCA(−), P63(+), GFAP(−), Oligo-2(−).

**Figure 6:**
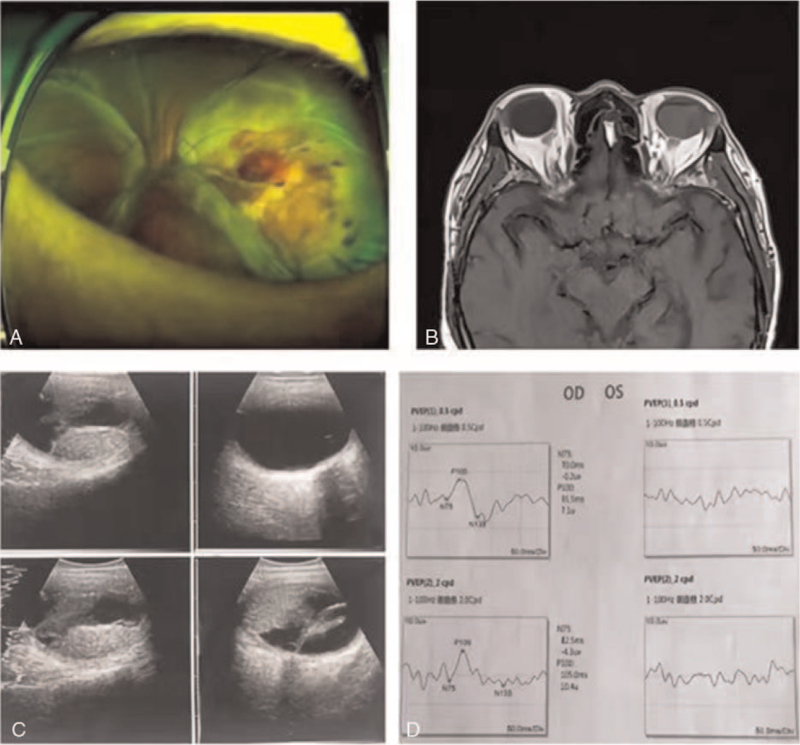
A) Three choroidal masses at the posterior pole of the left ocular fundus, macular involvement with subretinal fluid, without retinal tears, which appeared to represent retinal detachment. B) Solid homogenous mass rich in internal blood flow with exudative retinal detachment. C) The space-occupying lesions in the left eyeball appeared isointense on T1-weighted images and hypointense on T2-weighted images. D) There was no effective waveform at the peak of P100 in the left eye.

On March 30, 2020 the patient underwent craniotomy for the left parietal lobe tumour. The tumour was diagnosed as a metastatic poorly differentiated squamous cell carcinoma (Fig. 5). On May 7, 2020 the patient's visual acuity decreased again. Three choroidal masses at the posterior pole of the left ocular fundus, macular involvement with subretinal fluid, and no retinal tears, which appeared to represent retinal detachment. B-scan ultrasonography revealed 3 solid homogenous masses that were rich in internal blood flow, which were associated with exudative retinal detachment. MRI indicated space-occupying lesions in the posterior pole of the left eyeball. Ocular hemodynamics and pattern visual evoked potential analyses showed that there was no effective waveform at the peak of P100 in the left eye (Fig. 6). The patient was diagnosed with a CM. The patient was advised to undergo enucleation of the left eye, and the patient refused. Two months later, the patient died of systemic metastases. Informed consent was obtained from the patients’ families for the purpose of publication.

## 3 Discussion

CM accounts for 1% of all intraocular malignant tumours.^[6]^ Breast carcinoma, the origin of 40% to 53% of CM cases,^[7]^ is the most common primary source of CM, followed by lung cancer (20%–29%), and the source of 9% of primary lesions is unknown.^[8]^ CM from oesophageal cancer is rare and sporadic.^[9]^ The choroid is the most frequent site of metastasis in the orbital cavity because of its abundant blood supply, which may present a suitable microenvironment for malignant tumours, and the orbital and intraocular tissues lack lymphatic vessels, so the probability of lymphatic metastasis is lower than that of hematogenous metastasis.^[10]^ In some studies, the left eye was more frequently affected, and it has been proposed that this is related to a more direct path to the eye provided by the left common carotid, which originates from the aorta.^[11]^

Kodjikian et al^[12]^ believed that radiation therapy could cause eye inflammation. Invasion of the vitreous by inflammatory cells is likely to elevate the levels of vitreous proteases, which may degrade vitreous collagen. The loss of the gel structure occurs gradually, resulting in liquefaction. The fluid accumulates under the retina, and eventually, the retina detaches. Most studies have reported that retinal detachment caused by radiotherapy can result in retinal tears,^[13–15]^ and that retinal detachment usually occurs during radiotherapy,^[12,16,17]^ but the patient had completed radiotherapy 2 months earlier, so the possibility of radiotherapy causing retinal detachment is relatively low.

PTX and CDDP are mainly used to treat oesophageal cancer. Patients diagnosed with esophageal cancer have been reported to exhibit acute ocular neurotoxicity,^[18]^ visual field defects,^[19]^ papilledema, retrobulbar neuritis,^[20]^ and subretinal fluid accumulation after 10 days of PTX and CDDP administration at the recommended dose.^[21]^ It has been speculated that these effects may have been caused by dysfunctional retinal vascular regulation. Ophthalmologists may also consider exudative retinal detachment caused by the cytotoxicity of chemotherapy drugs, but choroidal metastatic carcinoma is often accompanied by exudative retinal detachment; therefore, the cause of the first retinal detachment is still unclear. In rare cases, choroidal metastases have preceded the diagnosis of primary carcinoma, and in a few cases, the lesions of primary carcinoma have been unclear, especially in male patients.

Choroidal metastases generally appear as creamy white or pale yellow masses^]^associated with subretinal fluid.^[4,5]^ B-scan ultrasonography examination shows flat or irregular solid space-occupying lesions with an uneven echo.^[22,23]^ MRI shows a well-demarcated choroidal mass that appears hyperintense on T1-weighted images and hypointense on T2-weighted images.^[24,25]^ FFA typically displays a hypofluorescent pattern in the early arterial phase and a hyperfluorescent pattern in the late phase.^[26]^ The lesions most similar to CM are choroidal melanoma and choroidal hemangioma; choroidal melanoma can be differentiated from CM by the larger size and “collar button”-like appearance of melanoma on B-scan ultrasonography as it spreads through the Bruch membrane.^[27]^ For choroidal haemangioma, the entire tumour body is hyperfluorescent in the early stage of FFA, and the blood vessels in the body are clearly visible.^[22]^ Later, the dye was quickly cleared, and the empty vessels showed mottled fluorescence. One of the characteristics of choroidal metastases is that they mostly occur in both eyes or in 1 eye as multifocal tumours.^[28]^

Retinal reattachment after 1 cycle of chemotherapy may be due to the inhibition of tiny choroidal metastases and the absorption of subretinal fluid after a certain course of chemotherapy. It has been reported in the literature that both gamma knife therapy and chemotherapy have therapeutic effects on choroidal metastases. Yang et al^[29]^ reported 2 patients with CM who were treated with pemetrexed (500 mg/m^2^) plus CDDP (75 mg/m^2^), and the choroidal tumour showed marked regression after the second cycle of chemotherapy. Singh et al^[30]^ also reported unilateral CM in the right eye of patients who received gemcitabine- and carboplatin-based chemotherapy for 6 cycles. The patient's condition improved, and no mass was found on fundus examination. In another patient with CM of breast cancer, CM did not change after 3 weeks of combined treatment with 5 kinds of chemotherapy drugs, but after 8 weeks of continuous chemotherapy, the exudative separation of the retina had completely resolved, and the choroidal tumour had flattened significantly.^[31]^ It was also reported in the literature that after gamma knife application to choroidal metastases, B-scan ultrasonography or MRI showed tumour size reduction with no adverse effects.^[32,33]^

These results suggest that it may take some time for chemotherapy drugs to inhibit choroidal metastases, and most of them require 2 cycles (1 cycle is 21 days) or longer. This finding is consistent with our findings. After 2 cycles of chemotherapy, metastasis was suppressed, and the retina was restored. The patient was treated with gamma knife therapy and chemotherapy again after the retina was restored, and the retina did not change significantly for 3 months. Therefore, the possibility that the first retinal reattachment was caused by chemotherapy was not excluded.

The second retinal detachment occurred after craniocerebral surgery, during which there was no chemoradiotherapy, and choroidal metastases may have progressed rapidly. There are 2 pathways by which brain tumour metastases can reach the choroid, including direct invasion through the skull and blood metastasis, but there was no obvious skull metastasis in this case. Therefore, the choroidal metastases may have been distant metastases of esophageal cancer or hematogenous metastases of brain tumours. The ophthalmic artery and internal carotid artery branch at a right angle, and the tumour cells in the blood are more likely to stay in the brain and have difficulty entering the eye. It is inferred that the primary cause of CM and brain metastasis is esophageal cancer. The brain mass was consistent with the pathological results of esophageal cancer; however, we could not examine the pathological results of CM because the patient refused to undergo enucleation. The literature has reported that the average survival time of patients with CM is approximately 1.9 to 4.3 months.^[34,35]^ Patients with choroidal metastases have a poor prognosis and a short life expectancy. The patient died 8 months after CM. The prognosis of the treatment strategy must be chosen carefully, and from 1970 to 2020, only 5 patients with CM of squamous esophageal cancer were reported.^[36]^ Two patients received radiotherapy with a prescribed dose of 30 to 50 Gy and received systemic chemotherapy at the same time. We believe that systemic chemotherapy and radiotherapy are effective treatment methods for CM of esophageal cancer, which should be recommended first.

## 4 Conclusion

In this case, esophageal cancer was the primary focus, and brain metastasis had occurred. Later, choroidal metastatic tumours were found with 2 retinal detachments, which are clinically rare. The specific mechanism remains unclear, and the possible causes include the cytotoxicity of radiotherapy and chemotherapy drugs as well as the exudative retinal detachment associated with microchoroidal metastases caused by esophageal cancer. A limitation of this study is that there are no reports of indocyanine green angiography and CM in pathological reports. There is no standard treatment plan for such cases, and further studies are needed in the future. Ophthalmologists should pay attention to the discovery of choroidal metastatic tumours in clinical work, comprehensively and carefully check the ophthalmology department, provide multidisciplinary early diagnosis, combined treatment and timely intervention, and improve the quality of life of patients.

## Author contributions

**Conceptualization:** Ying-Jun Li.

**Resources:** Jing-Hua Zhao, Yu-Jie Jia.

**Software:** Hong Cui, Hua Jin, Jian Zhao.

**Writing – original draft:** Shu-Hua Lin, Yong-Gen Xu.

**Writing – review & editing:** Shu-Hua Lin, Ying-Jun Li.
